# Development of a Nuclear Morphometric Signature for Prostate Cancer Risk in Negative Biopsies

**DOI:** 10.1371/journal.pone.0069457

**Published:** 2013-07-26

**Authors:** Peter H. Gann, Ryan Deaton, Anup Amatya, Mahesh Mohnani, Erika Enk Rueter, Yirong Yang, Viju Ananthanarayanan

**Affiliations:** 1 Department of Pathology, University of Illinois at Chicago, Chicago, Illinois, United States of America; 2 Department of Public Health Sciences, New Mexico State University, Las Cruces, New Mexico, United States of America; 3 College of Pharmacy, University of New Mexico, Albuquerque, New Mexico, United States of America; 4 Department of Pathology, University of Chicago, Chicago, Illinois, United States of America; University of Camp'nas, Brazil

## Abstract

**Background:**

Our objective was to develop and validate a multi-feature nuclear score based on image analysis of direct DNA staining, and to test its association with field effects and subsequent detection of prostate cancer (PCa) in benign biopsies.

**Methods:**

Tissue sections from 39 prostatectomies were Feulgen-stained and digitally scanned (400×), providing maps of DNA content per pixel. PCa and benign epithelial nuclei were randomly selected for measurement of 52 basic morphometric features. Logistic regression models discriminating benign from PCa nuclei, and benign from malignant nuclear populations, were built and cross-validated by AUC analysis. Nuclear populations were randomly collected <1 mm or >5 mm from cancer foci, and from cancer-free prostates, HGPIN, and PCa Gleason grade 3–5. Nuclei also were collected from negative biopsy subjects who had a subsequent diagnosis of PCa and age-matched cancer-free controls (20 pairs).

**Results:**

A multi-feature nuclear score discriminated cancer from benign cell populations with AUCs of 0.91 and 0.79, respectively, in training and validation sets of patients. In prostatectomy samples, both nuclear- and population-level models revealed cancer-like features in benign nuclei adjacent to PCa, compared to nuclei that were more distant or from PCa-free glands. In negative biopsies, a validated model with 5 variance features yielded significantly higher scores in cases than controls (*P* = 0.026).

**Conclusions:**

A multifeature nuclear morphometric score, obtained by automated digital analysis, was validated for discrimination of benign from cancer nuclei. This score demonstrated field effects in benign epithelial nuclei at varying distance from PCa lesions, and was associated with subsequent PCa detection in negative biopsies.

**Impact:**

This nuclear score shows promise as a risk predictor among men with negative biopsies and as an intermediate biomarker in Phase II chemoprevention trials. The results also suggest that subvisual disturbances in nuclear structure precede the development of pre-neoplastic lesions.

## Introduction

Subtle changes in nuclear shape, size and texture precede the histological recognition of prostate cancer (PCa) and thus might provide a useful biomarker indicating a field with high-risk benign tissue. Indeed, nuclear enlargement, irregularity, hyperchromasia and prominence of nucleoli are among the hallmarks used by pathologists to distinguish high-grade prostatic intraepithelial neoplasia (HGPIN), the most widely recognized premalignant lesion for PCa. More than 25 years ago, investigators with backgrounds in optical science and computing began using digital imaging techniques in an effort to transcend the limitations of the human eye and brain for recognizing and quantifying visual patterns in nuclei under the microscope [1]. These efforts reached a milestone when digital imaging was incorporated into the standard of care for cytological evaluation in cervical cancer screening. However, despite numerous reports of success using a variety of approaches and striking improvements in both hardware and software, computer-assisted nuclear morphometry still has abundant undeveloped potential for the discovery of useful biomarkers in PCa research [2], [3]. Veltri, et al. recently published an excellent review encompassing the history and evolution of this field [4].

In the present work we focus on quantification of nuclear DNA patterns as a biomarker for the early stage of pre-neoplastic change in benign prostatic epithelium, a stage associated with field effects or field cancerization [5], [6]. Validation of such a biomarker could lead to both clinical and research applications. Clinically, a morphometric profile could be used to predict the presence of cancer elsewhere in the gland in negative biopsies, and thus to inform decisions about monitoring and the need for repeat biopsy. PCa is the only common cancer that is typically diagnosed by random needle biopsy, due to the use of a serum test (PSA) as the chief indicator for biopsy and the absence of any imaging method for visualizing lesions. As a result, 70–75% of initial biopsies are negative and clinicians have no established basis for tailoring follow-up care, which could include monitoring of PSA and repeat biopsy. In terms of research application, a validated nuclear morphometric profile could serve as an intermediate endpoint biomarker for Phase II prevention trials, helping to identify the best candidate interventions for testing in lengthy and expensive Phase III studies.

We assembled a multidisciplinary group that included pathologists, epidemiologists, bioengineers, computer specialists and statisticians to develop an approach that would meet two basic requirements: 1) use of widely-available platforms for image acquisition and algorithm development, and 2) systematic validation. In this report we describe development of a continuous, multi-feature nuclear score based on pixel-by-pixel mapping of Feulgen DNA staining that accurately discriminates cancer and normal cell populations in prostate tissue and defines a field effect in high-risk benign areas.

## Methods

### Ethics statement

The project was reviewed and approved by the Institutional Review Board at the University of Illinois at Chicago. Tissue specimens were obtained under an IRB-approved waiver of consent applicable to de-identified samples of residual tissue not needed for clinical purposes. These procedures were in compliance with the privacy provisions of the Health Insurance Portability and Accountability Act (HIPAA) of 1996. The authors are open to collaboration involving sharing of the de-identified data, provided all local IRB requirements have been met.

### Tissue sample selection for model building and validation

We assembled two collections, from separate hospitals, of tissue blocks from radical prostatectomy patients with PCa. The first set, which was the learning set for developing models to discriminate cancer from benign nuclei, included 20 patients and the second set, used for external validation, included 11 patients. Among the 31 prostatectomy patients, 11 had cancers with Gleason sum grade 6, 10 with Gleason 7, and 10 with Gleason grades 8–9. All tissue blocks from 8 patients who underwent cystoprostatectomy for bladder cancer were also accessed. These wholly embedded prostates were devoid of PCa on serial sectioning at 3 mm intervals and were used to provide “supernormal” benign prostate.

### Feulgen staining

Tissue sections of 4μ thickness were placed on silanized glass slides and were stained using the Blue Feulgen Staining Kit (ScyTek Laboratories, Logan, UT). This stain uses the Feulgen reaction to directly bind dye to aldehyde groups in DNA that are exposed by treatment with hydrochloric acid. The amount of color developed is directly proportional to the amount of DNA in the stained nucleus; the stain has been validated for ploidy analysis. Serial sections were stained with hematoxylin and eosin so that key histological compartments could be easily identified on the single-color Feulgen slides. Adjacent sections from a single prostatectomy sample were included in each batch and the mean nuclear staining intensity was monitored to detect excessive inter-batch variation. Some nuclear morphometry studies have used the more routine hematoxylin and eosin (H&E) stain rather than Feulgen stain. Although the Feulgen stain is less familiar and somewhat more complex to perform, we believe that it has the important advantage of being roughly stoichiometric for DNA whereas the structures stained by H&E are non-specific. Moreover, we find that the Feulgen stain is easier to reproduce across multiple batches of samples.

### Image acquisition and processing

Slides were scanned at 400× on an Aperio ScanScope® CS whole-slide digital microscope (Aperio Inc., Vista, CA). Whole slide images were acquired using JPEG 2000 compression with the quality factor set at Q80 (20% loss from the raw image). A digital draw tool was used on the prostatectomy slides to demarcate areas of PCa by Gleason grade, HGPIN and benign areas within 1 mm or >5 mm from a cancer focus. Large scanned areas were divided into smaller subimage files (jpeg compression, quality factor 80) using the SnapShot Generator function in the Aperio Spectrum® image management software; these subimage files were exported to Matlab® (MathWorks, Inc., Natick, MA) for batch processing. The batch processed subimage files were 752×752 pixels in size (pixel size = 0.25μ^2^, approximately 400–600 pixels per nucleus), which was a manageable size for processing. Non-compressed tiff image files produced approximately 900–1000 pixels per nucleus; however, memory space requirements and throughput were substantially increased.

Customized routines in Matlab were used to identify pixels containing DNA and to segment individual nuclei using color-based K-means clustering and watershed algorithms. For the studies reported here, segmented nuclei were manually selected for morphometric feature collection using a graphical interface. Segmentation and nuclear selection was performed in several steps. First, each 752×752 pixel subimage was loaded into Matlab. Next, each image was converted from RGB to CIELAB (L*a*b*) colorspace (International Commission on Illumination, http://cie.co.at), whose three axes represent lightness value (L*), position on the red-green spectrum (a*), and position on the yellow-blue spectrum (b*). This reduces the number of color dimensions from three in RGB to two, the minimum required for the two-color white and blue Feulgen images. Next, a K-means clustering algorithm based on Euclidean distance was used to classify each pixel as blue (DNA) positive or white. The initial inputs for K-means clustering can affect the output; thus, initial L*a*b coordinates were set based on nuclei of good image quality as selected by a pathologist. Marker-controlled watershed segmentation based on gray-scale images was implemented in Matlab to define individual nuclear boundaries (http://www.mathworks.com/products/demos/image/watershed/ipexwatershed.html). This approach avoids over-segmentation by marking aggregates of intense pixels within the nuclei and background pixels outside of nuclei.

A trained technician was presented with a series of subimages with segmented nuclei on a PC monitor and mouse-clicked on each well-segmented nucleus after verifying its epithelial location, thus sending its morphometric data to an Excel spreadsheet. Most segmentation errors were due to over- or under-segmentation of touching or overlapping nuclei; among the selected nuclei we found no association between nuclear features and proximity to other nuclei, and thus we believe this procedure was relatively unbiased. A digital counter informed the technician when a total of 200 epithelial nuclei, from a wide range of subimages, had been selected from each whole section region of interest or biopsy. A total of 52 basic variables (see Table S1) were collected for each nucleus reflecting size, shape and DNA texture characteristics; more detailed descriptions of many of these features are available in the literature [7]. Special features that captured nuclear areas with either condensed or sparse DNA (“blobs” and “holes” in the Feulgen image) were developed based on identifying contiguous pixels with substantial deviations from the mean optical density (see Table S2). Other types of features, including fractal features, are readily calculated, but we did not use them in this analysis. Many features are highly correlated with each other and we found that, in general, expanding the library of features increased processing time while not substantially improving our results. To adjust for possible differences in staining across batches and to obtain common measurement units across features, we z-transformed each feature value by subtracting the mean and dividing by the standard deviation of that feature among all nuclei in the batch.

### Statistical analysis

The analysis centered on developing two types of models: one for discriminating individual cancer nuclei from benign nuclei, and the other for discriminating populations of cancer nuclei from benign populations. These models yielded multivariable scores we labeled as MFS_n_ (multifeature score, nuclear) and MFS_p_ (multifeature score, population) – corresponding to nuclear- and population-level scores, respectively. Using data on approximately 8,000 nuclei obtained from annotated PCa and benign areas in the learning set of 20 RP samples, we constructed logistic regression models to discriminate cancer nuclei (all Gleason grades) from benign nuclei at least 5 mm from a cancer focus. We compared several approaches for creating discriminatory multivariable models, including linear discriminant analysis, logistic regression, Support Vector Machines and neural networking and found, in agreement with earlier published work, that logistic regression was as good or better than other approaches [8]. Variables were selected for inclusion in the logistic models based on backwards elimination with an inclusion criterion of *P*<0.05. As expected, some variables were highly correlated, but no models failed to converge due to multicollinearity. The C statistic was calculated as the area-under-curve (AUC) for discriminating cancer from benign nuclei. A logistic model with 27 retained features provided a high AUC (0.93) in an independent test sample of benign and cancer nuclei obtained from the 11 cases in the external RP validation set. We used the two-sample Kolmogorov-Smirnov statistic to compare the distribution for MFS_n_ scores between nuclear populations sampled from various histological compartments in the 20 RP set, plus benign areas from the 8 prostatectomy cases with no significant PCa (i.e., “supernormal” nuclei).

We used two approaches to construct models for MFS_p_. In the first approach, which we called a two-step MFS_p_, we computed the MFS_n_ for each nucleus and then computed up to the fourth order moment (mean, standard deviation, skewness and kurtosis) of the MFS_n_ distribution for the population of nuclei obtained from each tissue sample (benign or malignant). These four summary variables were then used as predictors in logistic regression models for discriminating the benign vs. malignant populations and the fitted probability from the logistic model with a given set of covariates was designated as the MFS_p_. We derived a one-step MFS_p_ by calculating the mean, standard deviation, skewness and kurtosis for each nuclear feature from each tissue sample, yielding a total of 208 potential predictors (four times 52 basic features). We then used either backwards elimination or best-subset logistic regression in the training population of 28 patients to select a reduced set of predictors for discriminating cancer from benign nuclear populations. In the best-subset approach, the top 50 combinations of predictor sets with up to five variables were ranked based on the likelihood ratio chi square criterion. For each of those combinations we calculated the leave-one-out cross-validation AUC in the training set of samples, and the model with the highest AUC was chosen as the final model. The final regression weights for each predictor were computed as the average coefficient from all 28 trials in the training set. The final models chosen in the development process were then tested in the independent validation set that included 11 radical prostatectomy samples. Confidence limits for AUC (95% level) were computed using a nonparametric approach that exploits the properties of the Mann-Whitney statistic [9]. All statistical analyses were performed using SAS-PC, Version 9.1 (SAS, Inc., Cary, NC).

### External validation pilot study: case-control comparison of benign biopsies

As an external validation test, we compared populations of benign nuclei taken from negative prostate biopsies in which the patient was found to have PCa at least two years later (cases, n = 20) and benign nuclei from negative biopsies of patients who remained cancer-free (controls, n = 20). Cases and controls were matched on age and date of the index biopsy; all subjects were patients at the Jesse Brown Veterans Administration Medical Center in Chicago. Eligible controls had at least two negative biopsies after the index biopsy, no PSA>10 ng/ml, and no history of anti-hormonal therapy, including 5α-reductase inhibitors. We Feulgen-stained the negative index biopsy tissue and obtained nuclear morphometric features as described above. We then computed fitted MFS_p_ scores for each subject using both the backwards elimination and best-subset models previously derived from discrimination of benign and cancer cell populations in the prostatectomy samples. For both models, we calculated the AUC and 95% confidence limits for discriminating cases from controls, and performed a paired t-test for matched data.

## Results

**Figure 1:**
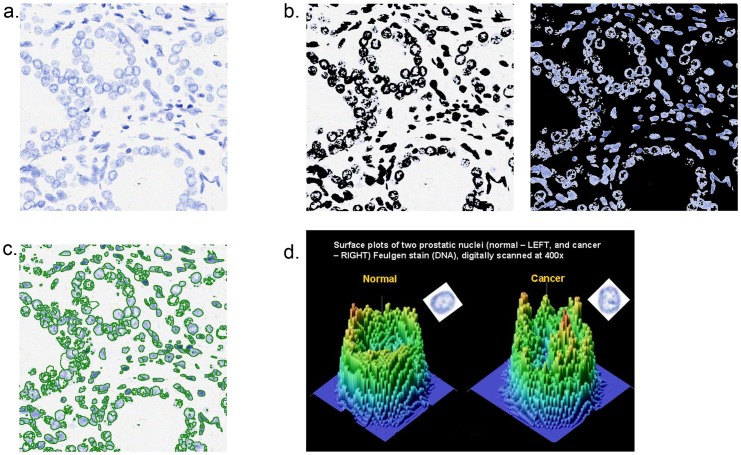
Process for obtaining nuclear morphometric data, showing a): a Feulgen-stain subimage, one of dozens obtained by breaking up whole slide images scanned at 400× on an Aperio ScanScope-CS®; b): a binary rendition of the previous image, produced by K-means clustering to identify pixels containing DNA; c): the same image with nuclei segmented using watershed algorithms available in Matlab®; d): three-dimensional maps showing optical density for DNA at each pixel in representative benign and PCa nuclei, respectively.

Figure 1 illustrates the process for obtaining pixel-by-pixel maps for each nucleus based on the optical density derived from DNA content. Whole slide scanned images of Feulgen-stained nuclei are broken into subimages (Figure 1a) each containing approximately 5.6K pixels. The associated binary image, created by K-means clustering, is shown in Figure 1b. Figure 1c shows the same subimage after watershed segmentation and indicates how well-segmented epithelial nuclei can be selected for measurement either manually or automatically. Figure 1d shows 3-dimensional plots of pixel maps for nuclei from benign and cancer areas, respectively.

**Figure 2:**
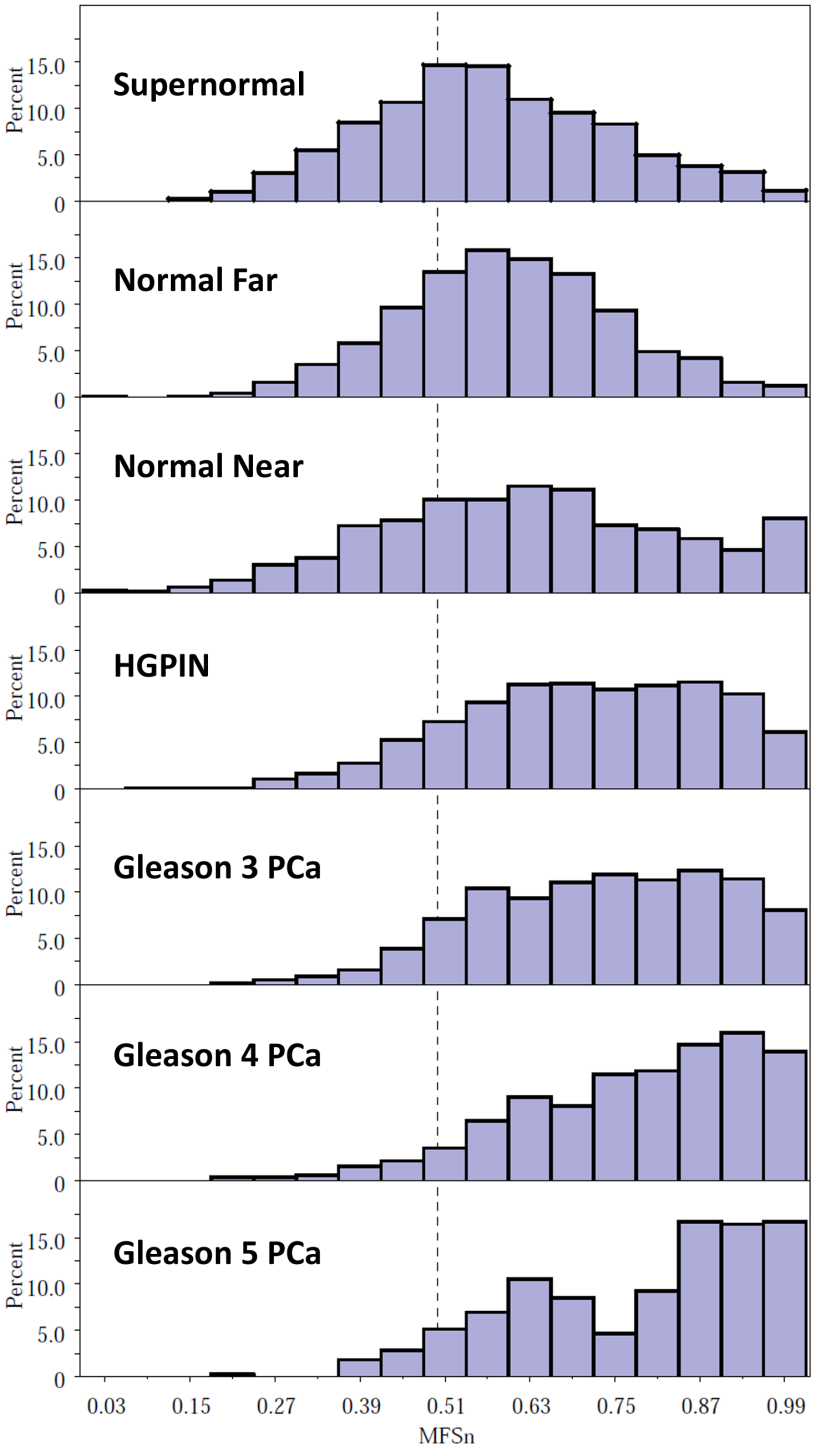
Frequency histograms of multifeature scores (MFS_n_) for nuclei from various malignant and benign tissue compartments. Fitted multifeature scores were generated for each nucleus from a logistic regression model comparing all cancer nuclei to normal-far nuclei (>5 mm from a cancer focus) from 20 prostatectomy specimens, with 27 covariate features selected by backwards elimination. Scores were calculated for populations of nuclei obtained from specific histological compartments in 20 RP and 8 cystoprostatectomy specimens. The frequency distributions for normal-far nuclei are significantly different from each other benign type (Kolmogorov-Smirnov D statistic <0.0001).

Figure 2 shows the relative frequency histograms for a nuclear-level multifeature score (MFS_n_) obtained from nuclei in various histological compartments from 20 RP and 8 cystoprostatectomy samples; each compartment is represented by at least several hundred nuclei. The logistic model used to generate MFS_n_ scores included 27 nuclear features and was based on discrimination between random PCa nuclei and benign nuclei distant from cancer (Normal Far) in the 20 RP cases. The MFS_n_, which is the anti-logged logit from the logistic model, is equivalent to the probability that a nucleus with a given set of feature values is a cancer nucleus, and thus ranges from 0 to 1.0. The frequency distribution of MFS_n_ shifts to the left as one progresses from Gleason 5 to Gleason 3 to HGPIN and continues to shift leftward for nuclei that are located near or far from a cancer focus, or are obtained from cancer-free prostates (supernormal). The frequency distributions for Normal Far nuclei (>5 mm from a PCa focus) are significantly different from both of the other benign types of nuclei (Kolmogorov-Smirnov D statistic *P*<0.0001).

**Figure 3:**
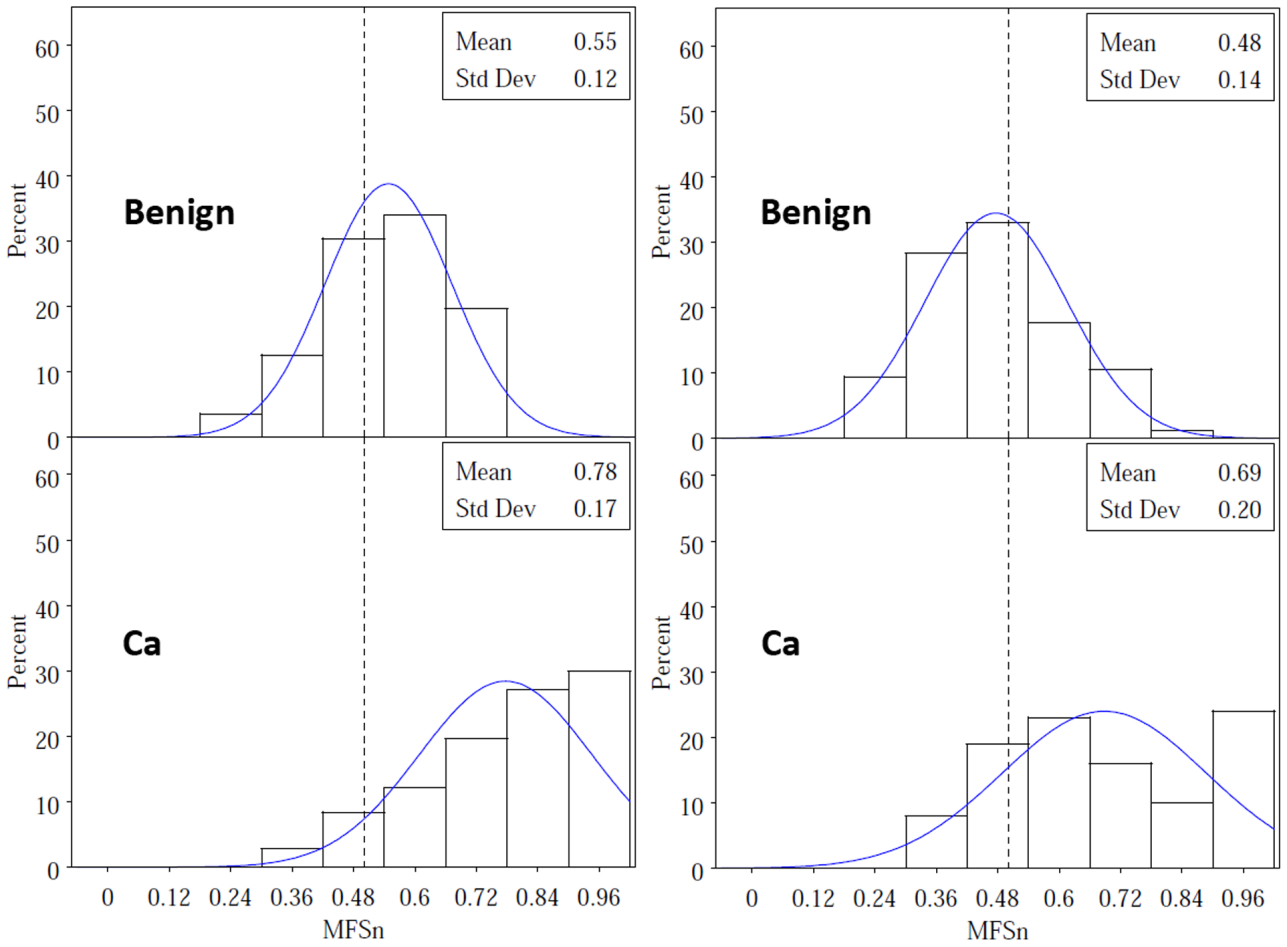
Frequency histograms for MFS_n_ benign and cancer nuclei from two selected subjects. MFS_n_ scores are shifted upward for cancer nuclei as expected; however, variance for MFS_n_ is also greater among cancer nuclei, reflecting pleomorphism.

**Figure 4:**
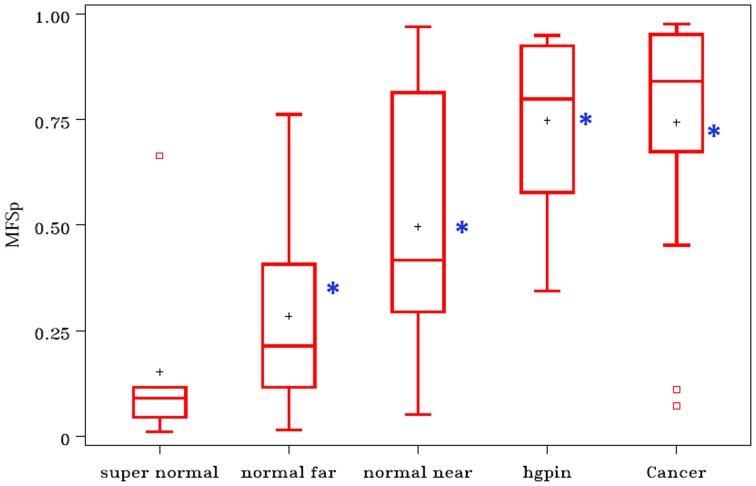
Boxplots for population-level multifeature scores (MFS_p_) from various tissue compartments in 20 radical prostatectomy subjects and 8 subjects with bladder cancer and supernormal prostates. The mean MFS_p_ scores for nuclear populations from the 11 validation RPs are shown by asterisks. The MFS_p_ scores were obtained from a logistic regression model with only two covariates: mean MFS_n_ and s.d. MFS_n_. MFS_n_ scores were generated from a 27-covariate logistic model with features selected by backwards elimination.

Typical frequency distributions of MFS_n_ for populations of benign and PCa nuclei from the same patient are shown in juxtaposition in Figure 3. The values for cancer nuclei are generally shifted to the right, but it is also clear that the variance in score is greater for cancer compared to benign nuclei. Population-level logistic models allow us to exploit this variance characteristic in discriminating cancerous from benign groups of nuclei. Figure 4 shows boxplots for MFS_p_ from two-step models with only two covariates: the mean and standard deviation of MFS_n_ for any given population of nuclei. Data in the boxplots come from the 20 RP subjects plus the 8 with cystoprostatectomy; the mean MFS_p_ scores for the 11 RP subjects in the validation set are represented by asterisks. The results indicate that Normal Near nuclear populations are intermediate between Normal Far and cancer, that Supernormal populations have lower scores than Normal Far, and that HGPIN nuclear populations are similar to cancer populations. The mean MFS_p_ scores for nuclei obtained from an external validation set of prostatectomy subjects with PCa confirm the same difference between Near vs. Far nuclei and the similarity between HGPIN and PCa nuclei.

Table 1 shows the AUCs for training and validation set discrimination of cancer cell from benign cell populations in prostatectomy specimens using two different models for computing MFS_p_. Model A, which used a conventional backwards elimination procedure to select the five best covariates, had a cross-validation AUC = 0.87 in the training set and AUC = 0.83 in the independent validation set. Model B, derived by comparing all possible subsets with five or fewer covariates based on leave-one-out cross-validation, had AUCs = 0.91 and 0.79 in the training and validation sets, respectively. The selected features and their standardized coefficients for the final backwards elimination and best subset MFS_p_ models (Models A and B, respectively) are shown in supplemental Table S3.

**Table 1:** A multifeature nuclear morphometric score (MFSp) accurately discriminates cancer vs. benign cell populations: AUC results for two one-step logistic regression models.

	Model A*		Model B**	
	AUC	95% CI	AUC	95% CI
Training set: (n = 28 cancer-benign pairs) leave-one-out cross validation	0.87	0.73–1.00	0.91	0.81–1.00
Validation set: (n = 11 cancer-benign pairs)	0.83	0.67–1.00	0.79	0.62–0.96

* Model A: Five features selected by backwards elimination: (FeretY_ave, MaxDiameter_ave, Elongation_ave, Slope_ave, ODKurtosis_ave).

** Model B: Five features (SumOD_sd, MaxDiameter_sd, TSD_sd, TEntropy_sd, No.MedDensityObjects_sd), selected after competition among all models with ≤5 covariates based on leave-one-out AUC.

We then compared populations of nuclei from matched pairs of negative biopsies in which the case subject subsequently had a diagnosis of PCa and the control remained cancer-free. Nuclear populations from each subject were assigned fitted MFS_p_ scores based on Model A and Model B; thus these models were developed using completely independent sample sets from prostatectomies rather than biopsies. The AUCs and paired T test results are shown in Table 2. Both models demonstrated significant differences between cases and controls, with cases having a more cancer-like nuclear morphometric signature.

**Table 2:** Comparing populations of benign nuclei from negative biopsies in a case-control study: subsequent diagnosis of prostate cancer is associated with a “cancer-like” nuclear morphometric score* (MFSp). Scores derived from external datasets.

	Model A*		Model B**	
	AUC	95% CI	AUC	95% CI
Case-Control Biopsies: Subsequent PCa vs. No PCa (n = 40 subjects; 20 pairs)	0.68	0.51–0.84	0.71	0.54–0.87
	**Paired t test ** ***P*** ** = 0.047**		**Paired t test ** ***P*** ** = 0.026**	

* Model A: 5 features selected by backwards elimination (FeretY_ave, MaxDiameter_ave, Elongation_ave, Slope_ave and ODKurtosis_ave).

** Model B: 5 features (SumOD_sd, MaxDiam_sd, TSD_sd, TEntropy_sd, No.MedDensityObjects_sd), selected after competition among all models with ≤5 covariates based on leave-one-out AUC.

## Discussion

In this study, we developed and validated a nuclear morphometric score, based on direct DNA staining, that accurately discriminated benign from cancer nuclei in prostate tissue. This score characterizes a field effect in histologically benign epithelial nuclei at varying distances from a cancer focus, and is associated with subsequent detection of PCa in an independent set of negative biopsies. It is significant to note that individual nuclear images were obtained with whole slide imaging at 400×. Thus, we were able to efficiently capture a large number of epithelial nuclei from each tissue sample at a magnification that allows for considerable detail regarding nuclear size, shape and DNA texture. Given pixel-level maps showing the spatial distribution of DNA within each nucleus, it is possible to generate an almost unlimited library of morphometric features. In the approach presented here, this agnostic library is mined to determine reduced sets of features for models that distinguish benign from malignant cells. Based on the assumption that there is a continuum in the evolution of nuclear shape and texture during carcinogenesis, the resulting multivariable scores provide a continuous index of the “cancer-ness” of each nucleus, and thus the collective “cancer-ness” for any population of nuclei. In general, the cancer-related features observed by digital analysis are simply subvisual extensions of nuclear changes that are well-recognized to the human eye under the microscope: cancer nuclei are somewhat larger and have a more clumped or irregular distribution of chromatin. Moreover, all of the features included in the most highly accurate model we found were related to the degree of variance among nuclei, thus supporting long-held views among pathologists regarding the importance of nuclear pleomorphism in cancer diagnosis.

Our study builds upon numerous earlier efforts that have applied digital nuclear morphometry to questions involving risk and prognosis in cancer of the breast [10], cervix [2], oropharynx/lung [11], colon [12], skin [13] and prostate. In the prostate, nuclear morphometry has been shown to detect abnormalities in benign tissue adjacent to cancer and HGPIN [14], [15]. Our data showed more evidence of a cancer phenotype in nuclei within 1 mm from a neoplastic lesion, compared to those at least 5 mm distant; however, earlier data suggests that these abnormalities might extend up to 10 mm from the border of a lesion [16]. Mairinger and co-workers physically extracted benign nuclei from paraffin-embedded tissue and, using Feulgen-stained cytospin preparations, reported that a combination of three chromatin texture features could accurately discriminate cases with PCa from those with only BPH [15]. Notably, two of these top three discriminatory features reflected inter-nucleus variation rather than mean values. Apart from the existence of a field effect in benign tissue, several studies have used nuclear morphometry of tumor and tumor-adjacent nuclei to discriminate subgroups of PCa patients according to the likelihood of progression while on active surveillance, PSA recurrence, metastasis or PCa-specific death [17], [18], [19], [20], [21]. Once again, variance features reflecting instability play a prominent role in these models, as they do in our results.

The biological mechanisms responsible for changes in nuclear structure that arise before the appearance of histologically recognizable neoplasia are not well understood. However, several processes could be implicated. First, the transition from loose euchromatin to more compact heterochromatin is an important mechanism for modulating gene expression that is controlled to a degree by covalent modification of histone tails. Patterns of global histone modification, by acetylation or methylation, are identifiable in PCa and have been associated with tumor aggressiveness [22], [23]. Mahmoud, et al observed decreased global acetylation at histone 3 lysine 9 (H3K9ac) in PIN and PCa compared to BPH tissue, and further observed similarities in H3K9ac expression between PIN and normal cells located near a PIN lesion [24]. There is also evidence that p300, a transcriptional co-activator of androgen receptor, can alter nuclear structure in prostate cells through its activity as a histone acetyltransferase or through its effects on the expression of nuclear matrix proteins such as lamin A and C [25]. Isharwal, et al. reported an association between specific nuclear morphometric features and p300 expression [26]. Irregularities in the nuclear envelope, including infolding and departures from a spherical shape, are a long-observed characteristic of PCa cells, yet the reasons for this irregularity and its functional significance are largely unknown [27]. Recent evidence indicates that expression of the MYC oncogene plays an important role in modulating nucleolar size, shape and number in the early phases of prostate carcinogenesis [28]. The role of the tumor microenvironment and paracrine signaling must also be considered, since a localized wound response can cause altered gene expression in benign stroma adjacent to PCa lesions [29]. These effects on the stromal field could induce subtle morphological changes in benign epithelia, including changes associated with epithelial-to-mesenchymal transformation [30].

This study adds to the field by systematically identifying prostate cancer-associated nuclear changes in benign epithelium using a widely available digital microscopy platform. Its strengths include validation with independent sets of radical prostatectomy and cystoprostatectomy samples, as well as a case-control analysis comparing negative biopsies from patients who either did or did not subsequently experience a PCa diagnosis. Given the large number of potential predictors for the one-step MFS_p_ and the relatively small number of subjects (28) in our training set, it is entirely possible that other combinations of features could have performed as well or better than those in our final model. Therefore, it is important to note that we used an efficient leave-one-out cross-validation approach (similar to bootstrap resampling) to select models and that our goal was not necessarily to find the absolute best model but to validate our chosen models in independent sets of images. The final models, which were derived from prostatectomy samples, not only produced risk scores that were associated with cancer in independent prostatectomy samples, but also demonstrated an association with cancer risk in biopsy specimens. Our technique deliberately excluded basal epithelial nuclei, and allowed for unbiased selection of a large number of luminal cell nuclei from each sample. Various approaches were compared for multivariable model development and were determined to be roughly equivalent.

Despite these strengths, the study has limitations as well. Our feature library may have included variables with some degree of collinearity, and although this would not affect predictive power, it would affect the ability to estimate the magnitude of associations for individual factors. Distinct feature classes for characterizing chromatin texture with lower redundancy should be added to the library, including fractal features, which have been associated with cancer prognosis in previous studies [31]. Importantly, while the results indicate that a multifeature score in negative biopsies is associated with subsequent PCa risk, this is not the same as demonstrating accurate prediction for individual subjects, as indicated by the relatively modest AUCs in Table 2. Development of a tool for clinical prediction will require further validation in larger, independent datasets using biopsy specimens. However, we note that even if the nuclear score fails to improve clinical prediction, a robust association with risk conveys important biological information about early steps in prostate carcinogenesis. Furthermore, accurate prediction at the individual level might not be necessary in order for this technique to serve as a useful intermediate biomarker in Phase II chemoprevention trials, where the objective is to identify potential agents with the greatest likelihood of efficacy.

The most important practical limitation to the method presented here involves the need for a human operator to select nuclei, which increases the time required to assemble an adequate collection for analysis from a tissue sample. The percentage of eligible epithelial nuclei that are selected for analysis with this operator-assisted approach is relatively low (we estimate this as 10–15%), but false positive nuclei are readily excluded while numbers are still quite adequate for analysis, and we took steps to minimize any bias during nucleus selection. In recent work, we have overcome this rate-limiting step by developing algorithms for automated selection of nuclei and have shown that the metrics from these nuclei are highly correlated with results obtained via manual selection from the same tissue samples. Future efforts will be devoted to studies exploring the biological basis for subvisual nuclear alteration in benign high-risk tissue, and to the testing of approaches that build discriminatory models on the direct comparison of high-risk vs. low-risk fields rather than on a cancer vs. benign comparison. We also plan to use nuclear morphometric profiling to evaluate the effects of chemopreventive agents on archived tissue from Phase II trials.

## Supporting Information

Figure S1 **Morphometric features based on areas of condensed or sparse DNA.** Further description of the approach used to define intra-nuclear areas of high or low DNA condensation (“blobs and holes”). Adapted from: Doudkine A, Macaulay C, Poulin N, Palcic B (1995) Nuclear texture measurements in image cytometry. Pathologica 87: 286–299.
(DOCX)

Table S1 **Nuclear morphometric features used in the analysis.** Descriptions of the 52 basic nuclear morphometric features contained in the feature library used for these studies. For analyses involving populations of nuclei, the mean, standard deviation, kurtosis and skewness values for each feature were calculated.
(DOCX)

Table S2 **Nuclear morphometric features selected for inclusion in two logistic regression models for discriminating populations of cancer vs. benign nuclei (one-step MFS_p_ score).**
(DOCX)

## References

[1] Diamond DA, Berry SJ, Umbricht C, Jewett HJ, Coffey DS (1982) Computerized image analysis of nuclear shape as a prognostic factor for prostatic cancer. The Prostate 3: 321–332. 7122329 10.1002/pros.2990030402

[2] Bacus JW, Boone CW, Bacus JV, Follen M, Kelloff GJ, et al (1999) Image morphometric nuclear grading of intraepithelial neoplastic lesions with applications to cancer chemoprevention trials. Cancer Epidemiol Biomarkers Prev 8: 1087–1094. 10613341

[3] Boone CW, Lieberman R, Mairinger T, Palcic B, Bacus J, et al (2001) Computer-assisted image analysis-derived intermediate endpoints. Urology 57: 129–131. 10.1016/s0090-4295(00)00956-0 11295610

[4] Veltri RW, Christudass CS, Isharwal S (2012) Nuclear morphometry, nucleomics and prostate cancer progression. Asian journal of andrology 14: 375–384. 22504875 10.1038/aja.2011.148 PMC3720156

[5] Nonn L, Ananthanarayanan V, Gann PH (2009) Evidence for field cancerization of the prostate. The Prostate 69: 1470–1479. 19462462 10.1002/pros.20983 PMC3690597

[6] Trujillo KA, Jones AC, Griffith JK, Bisoffi M (2012) Markers of field cancerization: proposed clinical applications in prostate biopsies. Prostate Cancer 2012: 302894. 22666601 10.1155/2012/302894 PMC3361299

[7] Doudkine A, Macaulay C, Poulin N, Palcic B (1995) Nuclear texture measurements in image cytometry. Pathologica 87: 286–299. 8570289

[8] Wolfe P, Murphy J, McGinley J, Zhu Z, Jiang W, et al (2004) Using nuclear morphometry to discriminate the tumorigenic potential of cells: a comparison of statistical methods. Cancer Epidemiol Biomarkers Prev 13: 976–988. 15184254

[9] DeLong ER, DeLong DM, Clarke-Pearson DL (1988) Comparing the areas under two or more correlated receiver operating characteristic curves: a nonparametric approach. Biometrics 44: 837–845. 3203132

[10] Frank DH, Kimler BF, Fabian CJ, Ranger-Moore J, Yozwiak M, et al (2009) Digital image analysis of breast epithelial cells collected by random periareolar fine-needle aspirates (RPFNA) from women at high risk for breast cancer taking hormone replacement and the aromatase inhibitor, letrozole, for six months. Breast Cancer Res Treat 115: 661–668. 19125322 10.1007/s10549-008-0274-0

[11] Ikeda N, MacAulay C, Lam S, LeRiche J, Payne P, et al (1998) Malignancy associated changes in bronchial epithelial cells and clinical application as a biomarker. Lung Cancer 19: 161–166. 9631363 10.1016/s0169-5002(97)00095-0

[12] Alberts DS, Einspahr JG, Krouse RS, Prasad A, Ranger-Moore J, et al (2007) Karyometry of the colonic mucosa. Cancer Epidemiol Biomarkers Prev 16: 2704–2716. 18086777 10.1158/1055-9965.EPI-07-0595

[13] Bartels PH, Yozwiak ML, Bartels HG, Liu Y, Hess LM, et al (2008) Limits of detection of chemopreventive efficacy: karyometry of skin biopsies. Cancer Epidemiol Biomarkers Prev 17: 1689–1695. 18583468 10.1158/1055-9965.EPI-08-0313 PMC2574734

[14] Montironi R, Hamilton PW, Scarpelli M, Thompson D, Bartels PH (1999) Subtle morphological and molecular changes in normal-looking epithelium in prostates with prostatic intraepithelial neoplasia or cancer. European urology 35: 468–473. 10325507 10.1159/000019881

[15] Mairinger T, Mikuz G, Gschwendtner A (1999) Nuclear chromatin texture analysis of nonmalignant tissue can detect adjacent prostatic adenocarcinoma. Prostate 41: 12–19. 10440871 10.1002/(sici)1097-0045(19990915)41:1<12::aid-pros3>3.0.co;2-#

[16] Bartels PH, Montironi R, Hamilton PW, Thompson D, Vaught L, et al (1998) Nuclear chromatin texture in prostatic lesions. II. PIN and malignancy associated changes. Analytical and quantitative cytology and histology/the International Academy of Cytology [and] American Society of Cytology 20: 397–406. 9801758

[17] Isharwal S, Makarov DV, Carter HB, Epstein JI, Partin AW, et al (2010) DNA content in the diagnostic biopsy for benign-adjacent and cancer-tissue areas predicts the need for treatment in men with T1c prostate cancer undergoing surveillance in an expectant management programme. BJU international 105: 329–333. 19678815 10.1111/j.1464-410X.2009.08791.x

[18] Veltri RW, Khan MA, Miller MC, Epstein JI, Mangold LA, et al (2004) Ability to predict metastasis based on pathology findings and alterations in nuclear structure of normal-appearing and cancer peripheral zone epithelium in the prostate. Clin Cancer Res 10: 3465–3473. 15161703 10.1158/1078-0432.CCR-03-0635

[19] Veltri RW, Miller MC, Isharwal S, Marlow C, Makarov DV, et al (2008) Prediction of prostate-specific antigen recurrence in men with long-term follow-up postprostatectomy using quantitative nuclear morphometry. Cancer Epidemiol Biomarkers Prev 17: 102–110. 18199716 10.1158/1055-9965.EPI-07-0175

[20] Veltri RW, Isharwal S, Miller MC, Epstein JI, Partin AW (2010) Nuclear roundness variance predicts prostate cancer progression, metastasis, and death: A prospective evaluation with up to 25 years of follow-up after radical prostatectomy. Prostate 70: 1333–1339. 20623633 10.1002/pros.21168

[21] Partin AW, Steinberg GD, Pitcock RV, Wu L, Piantadosi S, et al (1992) Use of nuclear morphometry, gleason histologic scoring, clinical stage, and age to predict disease-free survival among patients with prostate cancer. Cancer 70: 161–168. 1606538 10.1002/1097-0142(19920701)70:1<161::aid-cncr2820700126>3.0.co;2-5

[22] Ellinger J, Kahl P, von der Gathen J, Rogenhofer S, Heukamp LC, et al (2010) Global levels of histone modifications predict prostate cancer recurrence. Prostate 70: 61–69. 19739128 10.1002/pros.21038

[23] Seligson DB, Horvath S, Shi T, Yu H, Tze S, et al (2005) Global histone modification patterns predict risk of prostate cancer recurrence. Nature 435: 1262–1266. 15988529 10.1038/nature03672

[24] Mohamed MA, Greif PA, Diamond J, Sharaf O, Maxwell P, et al (2007) Epigenetic events, remodelling enzymes and their relationship to chromatin organization in prostatic intraepithelial neoplasia and prostatic adenocarcinoma. BJU international 99: 908–915. 17378849 10.1111/j.1464-410X.2006.06704.x

[25] Debes JD, Sebo TJ, Heemers HV, Kipp BR, Haugen DL, et al (2005) p300 modulates nuclear morphology in prostate cancer. Cancer Res 65: 708–712. 15705864

[26] Isharwal S, Miller MC, Marlow C, Makarov DV, Partin AW, et al (2008) p300 (histone acetyltransferase) biomarker predicts prostate cancer biochemical recurrence and correlates with changes in epithelia nuclear size and shape. The Prostate 68: 1097–1104. 18459105 10.1002/pros.20772 PMC3099408

[27] Fischer AH, Bardarov S Jr, Jiang Z (2004) Molecular aspects of diagnostic nucleolar and nuclear envelope changes in prostate cancer. J Cell Biochem 91: 170–184. 14689589 10.1002/jcb.10735

[28] Koh CM, Gurel B, Sutcliffe S, Aryee MJ, Schultz D, et al (2011) Alterations in nucleolar structure and gene expression programs in prostatic neoplasia are driven by the MYC oncogene. The American journal of pathology 178: 1824–1834. 21435462 10.1016/j.ajpath.2010.12.040 PMC3078425

[29] Jia Z, Wang Y, Sawyers A, Yao H, Rahmatpanah F, et al (2011) Diagnosis of prostate cancer using differentially expressed genes in stroma. Cancer Res 71: 2476–2487. 21459804 10.1158/0008-5472.CAN-10-2585 PMC3071046

[30] Cunha GR, Hayward SW, Wang YZ, Ricke WA (2003) Role of the stromal microenvironment in carcinogenesis of the prostate. Int J Cancer 107: 1–10. 12925950 10.1002/ijc.11335

[31] Metze K (2010) Fractal dimension of chromatin and cancer prognosis. Epigenomics 2: 601–604. 22122044 10.2217/epi.10.50

