# Supplementary material for: Development of a Nuclear Morphometric Signature for Prostate Cancer Risk in Negative Biopsies
Source: PLoS One. 2013 Jul 26;8(7):e69457. doi: 10.1371/journal.pone.0069457 (PMC3724855; doi:10.1371/journal.pone.0069457)

**Figure S1**

**Morphometric features based on areas of condensed or sparse DNA (“blobs” and “holes”)**

We created a set of discrete texture features using the approach proposed by Doudkine, et al (Doudkine A, Macaulay C, Poulin N, Palcic B. Nuclear texture measurements in image cytometry. Pathologica 87: 286-299, 1995). The average OD within each nucleus was computed and then various thresholds were defined to identify regions of condensed or sparse DNA. Regions of condensed DNA were defined as: > average OD and > average OD + 1 sd. Regions with sparse DNA were defined as: 1, 1.5, 2 or 2.5 sd below the mean OD. This resulted in 8 different combinations of upper and lower thresholds. Each set of thresholds was used to compute morphometric features shown in Appendix A (Low/Medium/High DNA Area, Low/Medium/High DNA Amount, Number of Low/Medium/High Density Objects, Compactness of Low/Medium/High Density Objects, and Asymmetry of Low/Medium/High Density Objects). The Figure below shows a side-on view of a nucleus with upper threshold at Average OD and lower threshold at Average OD – 2 sd. All contiguous regions above the upper threshold were defined as high density, and those below the lower threshold were defined as low density. Regions in between were defined as medium density.

To determine which thresholds for high and low density DNA were most useful, we compared a large random sample of benign and cancer nuclei for each blob and hole feature at each of the 8 threshold combinations. An upper threshold at (average OD + 1 sd) and lower threshold at (average OD – 1 sd) gave the greatest contrast between benign and cancer nuclei and this was used in further analyses.


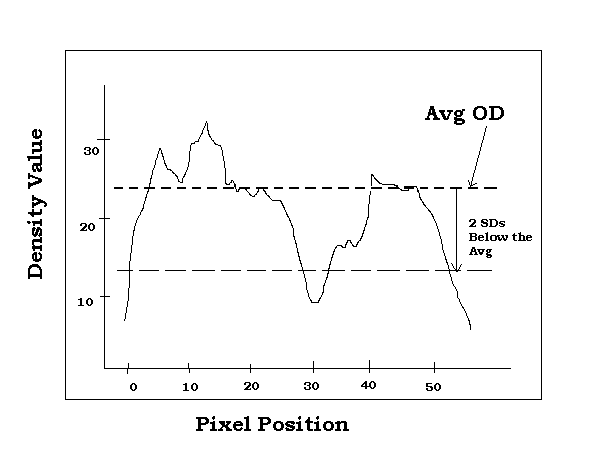

Supplement: Figure S1 — Morphometric features based on areas of condensed or sparse DNA. Further description of the approach used to define intra-nuclear areas of high or low DNA condensation (“blobs and holes”). Adapted from: Doudkine A, Macaulay C, Poulin N, Palcic B (1995) Nuclear texture measurements in image cytometry. Pathologica 87: 286–299. (DOCX) [file pone.0069457.s001.docx]
