# Supplementary material for: Development of a Nuclear Morphometric Signature for Prostate Cancer Risk in Negative Biopsies
Source: PLoS One. 2013 Jul 26;8(7):e69457. doi: 10.1371/journal.pone.0069457 (PMC3724855; doi:10.1371/journal.pone.0069457)
Supplement: Table S1 — Nuclear morphometric features used in the analysis. Descriptions of the 52 basic nuclear morphometric features contained in the feature library used for these studies. For analyses involving populations of nuclei, the mean, standard deviation, kurtosis and skewness values for each feature were calculated. (DOCX) [file pone.0069457.s002.docx]

| **Table S1. Nuclear morphometric features used in the analysis (n=52)** | | |
| --- | --- | --- |
| **Feature** | **Description** | **Source** |
| *Shape/size* | | |
| Area | *Area* = (number of pixels) x (pixel area) pixel area = 0.25 µ^2^ | Matlab IPT^a^ |
| Perimeter | *Perimeter* = length, in pixels, of boundary pixels | “ |
| Circularity | $\frac{{Perimeter}^{2}}{4\cdot\pi\cdot Area}$ | “ |
| Feret X | The width of bounding rectangular box around the nucleus (short side) | “ |
| Feret Y | The length of bounding rectangular box around the nuclear (long side) | “ |
| Maximum diameter | The maximum diameter of the nucleus, through the centroid | “ |
| Minimum diameter | The minimum diameter of the nucleus, through the centroid | “ |
| Elongation | Maximum diameter/Minimum diameter | “ |
| *Pixel intensity* | | |
| Sum optical density | The sum of each individual intensity value over all pixels comprising the nuclear body  $SOD= - \Sigma_{i}\Sigma_{j}log(\frac{I_{i,j}}{I_{0}})$ where I, j = pixel row, column | “ |
| Average optical density | *SOD/Area* = mean pixel intensity | “ |
| Maximum optical density | Nuclear pixel with maximum OD | “ |
| Minimum optical density | Nuclear pixel with minimum OD | “ |
| *Statistical moments from pixel intensity histogram: z_i_ = intensity, P(z) = histogram of intensity levels 0-255* | | |
| SD | Average contrast among pixels in the nucleus: $\sigma=\sqrt{\mu_{2}(z)}=\sqrt{\sigma^{2}}$ | Gonzalez RC, Woods RE^b^ |
| Symmetry | Measures skewness of the pixel intensity histogram; equals 0 for symmetric histograms: $\mu_{3}=\sum_{i=0}^{L-1} (z_{i}-m)^{3}p(z_{i})$ | “ |
| Kurtosis | Peakedness of pixel OD distribution; excess kurtosis relative to the normal distribution $\mu_{4}=\sum_{i=0}^{L-1} (z_{i}-m)^{4}p(z_{i})-3$ | “ |
| Smoothness | Measures relative smoothness of pixel intensity; R approaches 1 when intensity variation is high, approaches 0 when variation is low:  R = 1 -1/(1+σ^2^) | “ |
| Uniformity | Maximum when all gray levels are equal, indicating smoothness: $\sum_{i=0}^{L-1} p^{2}(z_{i})$  | “ |
| Entropy | Measure of randomness in intensity; larger value indicates coarser nucleus: $-\sum_{i=0}^{L-1} p(z_{i})\log_{2}p(z_{i})$ | “ |
| *Texture: pixel triplets^c^* | | |
| Valley | 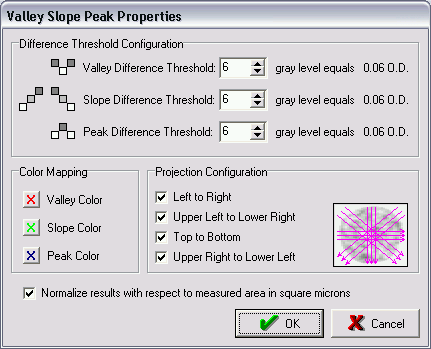The number of triplet pixels in the nucleus where the valley pixel OD is at least 6 OD greater than the two neighbor pixels. | Bacus JW. et al^d^ |
| Slope | 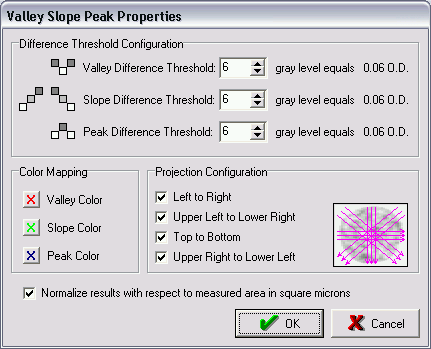 The number of triplet pixels in the nucleus where the change in OD between sloped pixels is at least 6 OD | “ |
| Peak | 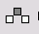 The number of triplet pixels in the nucleus where the peak pixel OD is at least 6 OD less than the two neighbor pixels | “ |
| Coarseness | Slope – (2*Peak – Valley) | “ |
| *Texture: Gray-Level Co-occurrence Matrix (GLCM) using a 3x3 matrix* | | |
| Sum mean | Mean sum of adjacent pixels, central pixel in 3x3 matrix vs. all neighbors (i.e., direct invariant): ∑ *Ps*(*i*) = *μs*  where *Ps(i)* = probability of sum intensity i; *µs* = sum-mean | Haralick RM, et al^e^ |
| Sum variance | Variance of the sum histogram for adjacent pixels: ∑(*i − μs*)^2^  *Ps*(*i*) | “ |
| Sum energy | Energy of the sum histogram; high values in images with larger regions of uniform intensity: ∑*Ps*(*i*) ^2^ | “ |
| Sum entropy | Randomness of the sum histogram; high values indicate more disorder in texture: ∑- *Ps*(*i*) log *Ps*(*i*) | “ |
| Difference mean | Mean of the difference histogram for adjacent pixels: ∑ *j P(d)* = *μd* | “ |
| Difference variance | Variance of the difference histogram: ∑ (*i − μd*)^2^  *Pd*(*i*) | “ |
| Difference energy | Energy of the difference histogram: ∑ *Pd* (*i*) ^2^ | “ |
| Difference entropy | Randomness of the difference histogram: ∑ - *Pd*(*i*) log *Pd*(*i*) | “ |
| Contrast | Intensity differences between neighboring pixels; increases with high magnitude of variation: ∑ *j ^2^ P(d)* | “ |
| Homogeneity | Opposite of contrast; high values indicate smooth texture with low variation: ∑ 1/ (1+j) ^2^ *P(d)* | “ |
| Correlation | Sum variance – contrast; indicates large regions of condensed chromatin with uniform intensity: ∑(*i − μs*)^2^  *Ps*(*i*) - ∑ *j ^2^ P(d)* | “ |
| Cluster shade | Similar to correlation; gives large positive values for light clumps against dark background and large negative values for dark clumps against light background: ∑ (*i − μs*)^3^  *Ps*(*i*) | “ |
| Cluster prominence | Another measure of chromatin condensation; large values associated with predominance of very high contrast clumps compared to background: ∑ (*i − μs*)^4^  *Ps*(*i*) | “ |
| Angular second moment | Sum energy times difference energy; strong measure of uniformity:  ∑ *Ps*(*i*) ^2 *^ ∑ *Pd* (*i*) ^2^ | “ |
| GCLM Entropy | Measure of randomness or disorder in the sum and difference histograms combined: ∑ - *Ps*(*i*) log *Ps*(*i*) + ∑ - *Pd*(*i*) log *Pd*(*i*) | “ |
| *Discrete texture features; areas of condensation and sparseness (“blobs and holes”)^f^* | | |
| Low DNA area | Fraction of total nuclear area occupied by low chromatin condensation | Doudkine, et al^g^ |
| Medium DNA area | “ “ “ medium “ “ | “ |
| High DNA area | “ “ “ high “ “ | “ |
| Low DNA amount | Ratio of integrated optical density in low density areas to total IOD | “ |
| Medium DNA amount | “ “ “ medium “ “ | “ |
| High DNA amount | “ “ “ high “ “ | “ |
| Low density objects | Number of spatially distinct objects with low density | “ |
| Medium density objects | “ “ “ medium “ | “ |
| High density objects | “ “ “ high “ | “ |
| Low DNA compactness | Compactness (circularity) of low density objects | “ |
| Medium DNA compactness | “ “ “ medium “ | “ |
| High DNA compactness | “ “ “ high “ | “ |
| Low center mass | Symmetry of optical density within low condensation areas | “ |
| Medium center mass | “ “ “ medium “ | “ |
| High center mass | “ “ “ high “ | “ |

^a^ Matlab Image Processing Toolbox (IPT), ver. R2013a, MathWorks, Inc., Natick, MA, 2013.

^b^ Gonzalez RC, Woods RE. Digital Image Processing (3^rd^ Ed.) Ch. 11, “Representation and Description”, Prentice Hall, N.J., 2007.

^c^ We varied the threshold for neighboring pixel difference in OD, comparing 2, 4, 6, 8 and 10 OD unit differences between a large sample of cancer and benign nuclei. Thresholds were set at 6 OD for the valley, slope and peak features used in final analyses.

^d^ Bacus JW, Grace LJ. Optical microscope system for standardized cell measurements and analyses. *Appl Optics* 26:3280-93, 1987.

^e^ Haralick RM, Shanmugam K, Dinstein I. Textural features for image classification. *IEEE Trans Systems Man Cybernetics* 3:610-21, 1973.

^f^ We compared a large random sample of benign and cancer nuclei for each feature at 8 threshold combinations. An upper threshold at (mean OD + 1 sd) and lower threshold at (mean OD – 1 sd) gave the greatest contrast between benign and cancer and this was used in further analyses.

^g^ Doudkine A, Macaulay C, Poulin N, Palcic B. Nuclear texture measurements in image cytometry. *Pathologica* 87:286-99, 1995.
