# Supplementary material for: Development of a Nuclear Morphometric Signature for Prostate Cancer Risk in Negative Biopsies
Source: PLoS One. 2013 Jul 26;8(7):e69457. doi: 10.1371/journal.pone.0069457 (PMC3724855; doi:10.1371/journal.pone.0069457)
Supplement: Table S2 — Nuclear morphometric features selected for inclusion in two logistic regression models for discriminating populations of cancer vs. benign nuclei (one-step MFSp score). (DOCX) [file pone.0069457.s003.docx]

| **Table S2. Nuclear morphometric features selected for inclusion in two logistic regression models for discriminating populations of cancer vs. benign nuclei (one-step MFS_p_ score)** | | |
| --- | --- | --- |
| **Feature name** | **Description** | **Standardized coefficient** |
| *Model A: predictors selected by backwards elimination* | | |
| Intercept | - | - 3.62 |
| FeretY_ave | longer axis of bounding box; mean | 3.82 |
| MaxDiameter_ave | maximum diameter through centroid; mean | - 8.88 |
| Elongation_ave | maximum divided by minimum diameter; mean | - 4.34 |
| Slope6_ave | number of pixel triplets on diagonal with >6 OD change in density; mean (see Appendix A) | 7.42 |
| ODKurtosis_ave | excess kurtosis relative to normal distribution for pixel OD distribution; mean | 12.24 |
|  |  |  |
| *Model B: predictors selected by ranking combinations based on leave-one-out AUC* | | |
| Intercept | - | - 5.05 |
| SumOD_sd | The sum of OD intensity value over all pixels comprising the nucleus; sd | 10.22 |
| MaxDiameter_sd | maximum diameter through centroid; sd | - 14.24 |
| TSD_sd | standard deviation of pixel intensity; sd | 12.06 |
| TEntropy_sd | measure of randomness of pixel intensity; sd | 11.48 |
| No.MedDensityObjects_sd | count of medium density regions within nucleus; sd | - 4.61 |
